# The Effects of Methylfolate on Cognitive Decline and Dementia: A Protocol for Systematic Review and Meta-Analysis

**DOI:** 10.3390/jcm12093075

**Published:** 2023-04-24

**Authors:** Leícia Iris de Assunção Prado, Ana Lúcia Junger, Leonardo Ferreira Caixeta, Matias Noll, Cesar de Oliveira, Érika Aparecida Silveira

**Affiliations:** 1 Postgraduate Program in Health Sciences, Faculty of Medicine, Federal University of Goiás, Goiania 74605-050, Brazil; 2 Goiano Federal Institute, Ceres 76300-000, Brazil; 3 Department of Neurology, School of Medicine, Federal University of Goiás, Goiania 74690-900, Brazil; 4 Departament of Sports Science and Clinical Biomechanics, University of Southernz Denmark, 5230 Odense, Denmark; 5 Department of Epidemiology & Public Health, Institute of Epidemiology & Health Care, University College London, London WC1E 6BT, UK

**Keywords:** folic acid, methylfolate, Alzheimer’s disease, dementia, ageing, cognitive function, mild cognitive impairment

## Abstract

Introduction: Dementia and cognitive decline are highly prevalent in later life and are growing public health concerns worldwide due to the increasing aging population. Individuals diagnosed with dementia have reduced mental function, resulting in social and cognitive skill deficits, increased dependence, and reduced autonomy—all of which are conditions associated with higher mortality rates. This systematic review aims to assess the effectiveness of treating dementia and cognitive decline with methylfolate. The main outcomes analyzed will be dementia and changes in cognitive function measured by different instruments in older adults treated with methylfolate. Secondary outcomes, such as inflammatory markers, plasma folic-acid levels, and total homocysteine, will also be assessed. Methods and Analysis: This study will be carried out in accordance with the Preferred Reporting Items for Systematic Reviews and Meta-Analyses guidelines. This protocol is registered in the International Prospective Registry of Systematic Reviews, under the code CRD42021275755. We will include clinical trials conducted with older adults diagnosed with dementia or cognitive decline and treated with folic acid. The searches will be conducted on the PubMed, Scopus, and Embase databases, and the articles will be selected by reading their titles and abstracts first, followed by the full text. The quality of the selected studies will be assessed using GRADE and the risk of bias will be assessed using the Downs-and-Black method. Ethics and Dissemination: The results will be presented at scientific conferences and disseminated by publishing a scientific article in an international English-language journal. We hope to find robust and significant evidence regarding the effectiveness of methylfolate supplementation in improving dementia symptoms and cognitive decline among older adults. By systematizing this evidence and possibly performing a meta-analysis study, we expect to significantly contribute to the treatment of this health problem, reduce mortality, and improve the quality of life and health of this population, boosting the development of medical protocols capable of reducing the financial effects of public health.

## 1. Introduction

With an ever-increasing aging population worldwide, a progressive increase in dementia cases has also been observed [1,2], since older adults are more susceptible to chronic diseases [3]. Alzheimer’s disease (AD) is the most prevalent cause of dementia [4]. Over the past 26 years, the global number of people living with dementia has increased from 20.2 million to 43.8 million, in 2016 [1]. Furthermore, the number of dementia cases is expected to triple by 2050, reaching approximately 152 million people globally [5].

Cognitive decline and dementia have become major public and clinical health problems due to their economic, social, and familial effects [5]. Individuals with dementia have reduced mental function, leading to deficits in social and cognitive skills [6], compromising the smooth performance of their basic and instrumental daily-living activities, which in turn leads to increased levels of dependence and reduced autonomy, as well as increased institutionalization rates and caregiver burden [7]. Cognitive decline and dementia are also associated with other comorbidities, such as femur fracture, dysphagia, congestive heart failure, urinary-tract infection, and hypertension [3]. Furthermore, chronic diseases, such as diabetes, cardiovascular disease, depression, and inflammatory bowel disease may be associated with an increased AD risk [8,9]. Individuals with dementia are more fragile and have higher mortality rates [10,11].

Given the increasing prevalence of dementia and its impact on health and mortality, several studies have identified factors that can contribute to the treatment of or reduction in the damage caused. A recent systematic review of prospective observational studies and randomized clinical trials, considered one of the most comprehensive studies on AD prevention, proposes several clinical suggestions for AD-prevention measures, including the practice of stimulating mental activities, a healthy lifestyle, and others [12].

Regarding the mechanisms involved in the etiology and pathogenesis of brain damage induced by dementia, some studies have documented the importance of nutritional aspects [6,13]. Among these, deficiency in folate, also known as methylfolate, folic acid, or vitamin B9, appears to contribute to increased blood and intracellular levels of homocysteine (Hcy) [14]. These, in turn, have been associated with impaired cognitive function and neurodegenerative disorders, such as AD [15] and vascular dementia [16].

High intraneuronal levels of total homocysteine (tHcy) lead to inadequate methylation, which can disrupt brain metabolism and cause cognitive impairment [17]. The underlying mechanisms of tHcy as a risk factor for vascular dementia and AD remain unclear, although many of the ways in which tHcy can damage neurons have been demonstrated, including by causing endothelial dysfunction, cerebral microangiopathy, and increased oxidative stress [6]. Increased levels of tHcy, also known as hyperhomocysteinemia, contribute to excitotoxicity, a process in which neurotransmitter receptors (glutamate) are hyper-stimulated, damaging neural cells and favoring oxidative stress [18]. Thus, analyses of this biomarker are often included in studies using methylfolate supplementation.

Folate deficiency leads to the incorporation of nitrogenous bases in DNA, impairing repair mechanisms in neuronal cells and sensitizing them to oxidative damage and beta-amyloid toxicity [19,20]. These inflammatory processes contribute to the development of neurodegenerative diseases [14], including AD, which induces vascular neurotoxicity [21,22]. Clinical trials conducted on older adults indicated that oral supplementation with folic acid improved cognitive function in relation to daily-life activities, mental condition, and inflammatory markers, such as interleukin-6 (IL-6) and C-reactive protein (CRP) [23,24,25]. Other clinical trials on older adults revealed more pronounced improvements in cognitive functions, such as individuals’ mental condition and ability to perform daily activities, when methylfolate was used as a supplement in combination with other vitamins, such as vitamin B12 (cobalamin) and vitamin B6 (pyridoxine), compared to when it was used alone [4,26,27]. In contrast, some clinical trials failed to show any signs of improvement in cognitive function with methylfolate supplementation alone or in combination with other vitamins, such as B12 and B6 [28,29,30]. 

In addition to the clinical trials assessing the efficacy of methylfolate in older adults, numerous systematic reviews and meta-analyses have been conducted on this subject [31,32,33,34]. Two recently published meta-analyses on folate differ from our study in that one [35] had cerebrovascular disease as its endpoint, while we will evaluate all types of dementia, and the other [36] focused on observational studies, while we intend to examine the effect of methylfolate interventions, and will only include randomized controlled trials (RCTs). Moreover, these studies were unable to demonstrate the beneficial effects of methylfolate or folic-acid supplementation on older adults with cognitive impairment, either when administered alone or when used in combination with nutritional approaches, and regardless of the route of administration [31,32,33,34]. Given the conflicting findings and the gaps in knowledge regarding the effectiveness of folic acid in reducing cognitive improvement, whether in CCL or in forms of dementia including mild cognitive impairment (MCI), it is important to summarize the evidence on these pharmacological and non-pharmacological interventions [36,37].

Non-pharmacological interventions are relatively new. However, they show promise in slowing the progression of dementia or MCI. Psychosocial interventions [35] and personalized nutritional strategies can be effective alternatives to improve both the treatment and prevention of dementia [36,37]. Given the urgent need to develop new and effective therapeutic interventions in the battle against AD, a study that gathered significant evidence on the effectiveness of methylfolate in reducing cognitive deterioration would be highly relevant and may contribute to improving the treatment and care of older adults with cognitive decline and dementia. Therefore, this systematic review aims to assess the effectiveness of treating dementia and cognitive decline with methylfolate.

## 2. Methods

### 2.1. Protocol and Registration

This systematic review will be conducted and reported according to the criteria of the Preferred Report Items for Systematic Review and Meta-Analysis Protocols (PRISMA) [38]. This protocol was written using scientific writing methods [39] to improve the technical quality of the article.

We will adopt the PICO structure [40], which is defined as follows: population (P) consists of individuals of both sexes diagnosed with mild cognitive deficits and dementias; intervention (I) consists of methylfolate supplementation (folic acid, vitamin B9, or folate), comparison (C) corresponds to control groups left without methylfolate supplementation, were given placebo, or underwent different intervention times; and outcome (O) represents a reduction in cognitive deficits.

This protocol is registered in the International Prospective Registry of Systematic Reviews under the code CRD42021275755. Any changes made to this protocol during this study will be reported in PROSPERO and in the final version of the manuscript.

### 2.2. Definitions

Mild cognitive impairment (MCI) is a transitional stage between intact cognition and dementia [41]. This category includes individuals whose cognitive deficits are beyond those typically observed during ageing [31]. These cognitive changes should be mild enough for there to be no evidence of significant impairment in social or occupational functioning [42]. In general, MCl is a state in which individuals have subjective and objective memory impairment inconsistent with age, but with overall cognitive function and performance in normal cognitive domains [31]. Thus, the main criteria that distinguish MCI from dementia are the preservation of independent functional abilities related to daily activities and instrumental activities of daily living, and a lack of significant impairment on social or occupational functioning [43].

To assess whether an individual is cognitively normal or presents with a certain degree of cognitive impairment, studies have used validated cognitive-rating scales in a more practical and versatile manner [44]. Many validated clinical neuropsychological measures are available to assess these cognitive domains, including scales such as the Mini Mental State Examination (MMSE) [45], the Montreal Cognitive Assessment for Global Cognition [46] and the Daily Living Questionnaire [47], among others. All these tools have been used for the evaluation of functionality.

Dementia: Dementia is a clinical syndrome characterized by cognitive and functional decline. It is usually progressive and involves the impairment of cognitive functioning, with memory being the most frequent domain affected in the early stages. Other higher cortical functions, such as orientation, understanding, learning, language, and judgment are also frequently affected. In most cases, the onset and subsequent progression of dementia are gradual. As the syndrome progresses, people with dementia may become dependent on others for support in all activities of daily living [31].

When cognitive impairment is sufficiently significant to interfere with daily functioning, a patient is diagnosed with dementia [42]. Dementia is a decline in cognitive functions, such as thinking, understanding, and decision-making, which progressively disturbs independent performance of activities of daily living. It occurs in different diseases, such as metabolic diseases, nutritional deficiencies, and neurodegenerative diseases such as AD, frontotemporal dementia, vascular dementia, and Lewy-body dementia [48,49,50].

In all subtypes of dementia, such as AD, frontotemporal dementia, vascular dementia, Lewy body dementia and mixed dementia, patients present with cognitive decline.

Dementia will be diagnosed according to the criteria of the Diagnostic and Statistical Manual of Mental Disorders at the time of this study. Alzheimer’s Disease will be assessed using the National Institute of Neurological and Communicative Disorders and Stroke and the Alzheimer’s Disease and Related Disorders Association [51] criteria, the criteria of working group of the National Institute on Aging and the Alzheimer’s Association [52], or other validated criteria. Other causes of dementia will be diagnosed based on several globally accepted criteria.

This study intends to investigate, as a primary outcome, cognitive function in older adults diagnosed with some type of dementia or MCI receiving methylfolate supplementation. As instruments for measuring cognitive function that were commonly applied in recent studies, the MMSE, Scale-Revised (WAIS-RC, Informant questionnaire on cognitive decline, Alzheimer’s Disease Assessment Scale (ADAS), ADAS-cog, and Activities of Daily Living scales can be used. However, results from other instruments measuring cognitive function found during the exploration of the articles included in this research will also be analyzed.

As additional markers of cognitive function, we will analyze blood vitamin levels, such as folic acid (vitamin B9), cobalamin (vitamin B12), and pyridoxine (vitamin B6), in addition to inflammatory markers, such as IL-6, CRP, tumor necrosis factor, interferon, amyloid beta (Aβ), and serum or plasma levels of tHcy.

### 2.3. Research Strategy

Selected articles must have been published in English, Spanish, or Portuguese, with no restrictions on the publication date. The search will begin with December 2002.

The PubMed, Embase, LILACS, and Scopus databases will be accessed. The bibliographies from included studies will be searched by hand for additional research. Google Scholar will not be used. The terms will be searched in a standardized way in the three databases according to the following combination: (dementia OR “dementia vascular” OR “frontotemporal dementia” OR “Alzheimer disease” OR “neurocognitive disorders” OR “cognition disorders” OR amnesia OR “neurodegenerative diseases” OR “neurodegenerative disease” OR “cognitive dysfunction” OR “cognitive impairments” OR “Alzheimer dementia” OR “vascular dementia” OR “Lewy body disease” OR “Lewy body dementia” OR ““Cognitive function” OR cognition) AND (“Clinical Trial” OR “Randomized Controlled Trial” OR “Controlled Clinical Trial” OR “controlled trial” OR trial OR “double blind procedure” OR “Double blind method” OR “crossover procedure” OR “Cross-over studies”) AND (“folic acid” OR “pteroylpolyglutamic acids” OR tetrahydrofolate OR “vitamin b9” OR “formyltetrahydrofolate” OR methylfolate “L-methylfolate”). In the case of PubMed, MeSH terms will also be used to perform a broader search (complete search strategy and search results are shown in Table 1, Table 2, Table 3, Table 4 and Table 5). 

The search strategy will be complemented by a manual search of the reference list of the included studies.

### 2.4. Study Population

Older adults (aged ≥60 or ≥65 years, depending on the classification used where the research was conducted) diagnosed with MCI or some type of dementia. Individuals who live in the community and those who are in hospitals/institutions, as well as outpatients, will be eligible for inclusion in the study. Individuals diagnosed with liver diseases, cancers, malabsorptive diseases, or chronic kidney disease (on dialysis) will not be selected, in accordance with the exclusion criteria.

### 2.5. Criteria and Eligibility

#### 2.5.1. Inclusion Criteria

- Older adults (aged ≥60 or ≥65 years, depending on the classification used where the research was conducted) with a diagnosis of MCI or some type of dementia.
- Randomized and controlled clinical trials based on methylfolate or folic-acid supplementation, regardless of the type of administration route, dose used, and intervention time.
- Representative population samples and a cohort of individuals in hospitals, health centers, outpatient clinics, or institutionalized settings, i.e., nursing homes.
- Studies published in English, Portuguese, or Spanish.

#### 2.5.2. Exclusion Criteria

Publications on the topic under investigation in the form of opinion articles, letters to the editor, comments, editorials, systematic reviews, case reports, protocols, observational studies and those with qualitative data, including:

- Studies whose main data are not accessible, even upon author request.
- Duplicates: for studies published in more than one article, we will consider the most comprehensive, with the largest sample sizes.
- Studies carried out with indigenous individuals.
- Studies conducted on individuals diagnosed with liver disease, chronic kidney disease (on dialysis), cancers, malabsorptive diseases such as inflammatory bowel disease, celiac disease or other intestinal diseases that interfere with oral interventions, chronic infections, bipolar affective disorder, Parkinson’s disease, multiple sclerosis, motor neuron disease, developmental impairment, central-nervous-system inflammation, progressive malignancy, psychotic symptoms, schizophrenia, alcohol or other drug addiction, and any medical or psychological condition preventing them from completing the ratings.
- Studies with individuals who have used nutritional supplements known to interfere with nutritional status, folate metabolism, or cognitive function three months prior to recruitment.

### 2.6. Reviewer Training

The authors in charge of eligibility and article-selection criteria (LIAP and ALJ) will be trained to apply the inclusion and exclusion criteria. Following training, LIAP and ALJ will complete the selection process by reading the titles and abstracts.

Potential disagreements regarding this eligibility step are to be resolved by EAS. The included studies will then be read in full for the final selection and data-extraction processes. Reviewers will also receive training in the application of the instruments to assess the risk of bias. The selection steps are to be performed using Endnote and Rayyan software [53].

#### Review Process

The search for articles will be performed by two independent researchers (LIAP and ALJ) and a third senior reviewer (EAS). Articles will be included after reading their titles and abstracts. Finally, the full content will be analyzed for inclusion.

After the search step, articles will be grouped, and duplicates will be removed using the Mendeley software. Two reviewers (LIAP and ALJ) will then independently screen the titles and abstracts of all articles identified in the literature search for inclusion. Disagreements regarding inclusion will be discussed and resolved by a third reviewer (EAS). The screening process will be performed by both reviewers using Rayyan software [53].

The reliability among evaluators in rating individual components will be determined by calculating the percentage of agreement and Cohen’s kappa [54]. The remaining articles will be read and evaluated to determine their eligibility based on the inclusion and exclusion criteria. Finally, eligible articles will be included in the systematic review.

**Figure 1:**
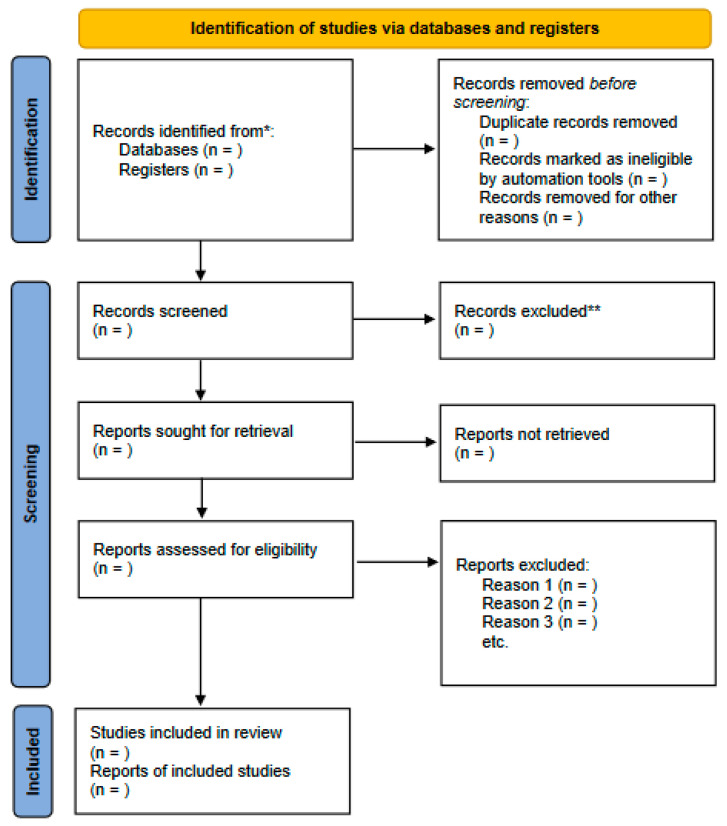
Flowchart of the screening process for this systematic review. * Consider, if feasible, reporting the number of records identified from each database or register searched (rather than the total number across all databases/registers). ** If automation tools were used, indicate how many records were excluded by a human and how many were excluded by automation tools.

In addition, the reference lists of the included articles will be searched to identify additional studies that may have been missing during the database search. The process of selecting articles for the systematic review will be completed by the end of July 2023. The flowchart for this systematic review, with information on the screening process for these studies, is shown in Figure 1.

### 2.7. Data Extraction

Data will be extracted by two independent reviewers (LIAP and ALJ) using a standardized form prepared by the authors. The following aspects will be considered: author, year, location, study population (age group, sample size, sex), study design, cut-off point (baseline), technique, intervention (type, dosage, duration, folic acid, isolated or combined with other substances), control, type of cognitive impairment, type of cognitive measurement tool, and primary outcomes.

Potential disagreements between reviewers while screening the studies will be resolved by a senior reviewer (EAS). We plan to contact the authors of the articles to request missing or additional data when necessary.

### 2.8. Assessment of the Quality of the Articles and Risk of Bias

To assess the quality of the findings in the included articles, the Grading of Recommendations, Assessment, Development and Evaluations (GRADE) system will be used [55], estimating for each article one of the four quality-level scores, i.e., high, moderate, low, and very low.

The risk of bias will be assessed using the Downs-and-Black instrument [56], which consists of a score that aims to assess the methodological quality and was specially designed to include randomized and non-randomized studies. It features 27 scoring items. A summarized quality score (0–27 points) for each study will be calculated, expressing the number of items in accordance with the total percentage. Scores >70% will be used to define low risk of bias.

### 2.9. Synthesis of Evidence and Statistical Analyses

If the data extracted from different studies are homogeneous, they will be combined in a meta-analysis.

The effects of folic-acid supplementation on outcomes will be analyzed according to the odds ratio, linear regression, and mean reduction reported in the studies.

A subgroup analysis will be performed using different routes of administration of folic-acid supplementation, durations of interventions, and treatment dosages. If data are sufficient, we will analyze the subgroups regarding the demographic data of the participants, i.e., sex, age group, and status of hospitalization.

Patient and Public Involvement

No patients involved.

## 3. Discussion

This systematic-review protocol will shed light on a relevant public health concern, which increasingly affects individuals aged 60 and older and has serious health implications. This study will also provide an overview of the theoretical framework for the potential use of therapeutic approaches involving methylfolate or folic-acid supplementation to improve cognitive function in individuals with MCI or dementia.

The small number of published original studies with clinical-trial designs lends support to the publication of a systematic review of the investigations on the subject, since there are currently limited conclusions regarding the impact of methylfolate supplementation on cognitive impairment in individuals diagnosed with MIC or dementia [31,34,57]. As pointed out above, two recent meta-analyses on folate have been published. However, they have different goals from those of our study: one meta-analysis [58] has cerebrovascular disease as an outcome and the other [59] focuses on observational studies. Our study will not be limited to cerebrovascular diseases; we intend to analyze the effect of methylfolate interventions on all types of dementia, with a focus on RCTs. The use of various cognitive-assessment tests and scales, different doses of methylfolate supplementation, a short intervention period, and the combination of other vitamins/substances are the most frequently cited limitations in the reviews, yielding conflicting conclusions regarding the possible benefits of using methylfolate to treat cognitive decline in individuals with MCI and dementia.

After all the steps of this systematic review and potential meta-analysis, we expect to be able to demonstrate the effect of methylfolate supplementation on improving the cognitive functioning of patients with MCI and dementia. Furthermore, we intend to establish whether methylfolate supplementation reduces inflammatory markers and tHcy levels in individuals with MCI and several types of dementia. From a cognitive perspective, determining and understanding the effectiveness, through scientific studies, of methylfolate or folic-acid supplementation can contribute to improving clinical treatment protocols, as well as helping to develop up-to-date recommendations and clinical protocols, such as the best dose and the most suitable time for its administration, in the treatment of cognitive impairment in individuals affected by MCI or dementia. In addition, the results of this review may contribute to reducing the impact on health costs and identifying key needs to be addressed in future research.

This systematic review may feature some limitations. First, it might not generate sufficient data for a meta-analysis. Second, there are few publications that specifically investigate methylfolate supplementation, with an emphasis on the outcomes of interest of this research, in a representative population sample.

## Figures and Tables

**Table 1:** Blocks of the main terms used in the search strategy.

1.	Dementia OR Cognitive Impairments OR Cognitive disorders
2.	Clinical Trial OR Randomized Controlled Trial OR Controlled Clinical Trial
3.	Methylfolate OR Folic acid OR Vitamin B9
4.	1 AND 2 AND 3

**Table 2:** Search strategy for PubMed.

	PubMed	Results
1.	(((((((((((((((((((((((((((((dementia [MeSH Terms]) OR (“dementia, vascular” [MeSH Terms])) OR (“frontotemporal dementia” [MeSH Terms])) OR (“alzheimer disease” [MeSH Terms])) OR (“neurocognitive disorders” [MeSH Terms])) OR (“cognition disorders” [MeSH Terms])) OR (amnesia [MeSH Terms])) OR (“neurodegenerative diseases” [MeSH Terms])) OR (“cognitive dysfunction” [MeSH Terms])) OR (“Lewy body disease” [MeSH Terms])) OR (cognition [MeSH Terms])) OR (dementia [Title/Abstract])) OR (“dementia vascular” [Title/Abstract])) OR (“frontotemporal dementia” [Title/Abstract])) OR (“alzheimer disease” [Title/Abstract])) OR (“neurocognitive disorders” [Title/Abstract])) OR (“cognition disorders” [Title/Abstract])) OR (amnesia [Title/Abstract])) OR (“neurodegenerative diseases” [Title/Abstract])) OR (“neurodegenerative disease” [Title/Abstract])) OR (“cognitive dysfunction” [Title/Abstract])) OR (“cognitive impairments” [Title/Abstract])) OR (“alzheimer dementia” [Title/Abstract])) OR (“vascular dementia” [Title/Abstract])) OR (“Lewy body disease” [Title/Abstract])) OR (“Lewy body dementia” [Title/Abstract])) OR (“Cognitive function” [Title/Abstract])) OR (cognition [Title/Abstract])) OR (“Cognitive Symptoms” [Title/Abstract])) OR (“Cognitive Symptom” [Title/Abstract])	754,959
2.	(((((((((((“Double blind method” [MeSH Terms]) OR (“Cross-over studies” [MeSH Terms])) OR (“Clinical Trial” [Title/Abstract])) OR (“Randomized Controlled Trial” [Title/Abstract])) OR (“Controlled Clinical Trial” [Title/Abstract])) OR (“controlled trial” [Title/Abstract])) OR (trial [Title/Abstract])) OR (“double blind procedure” [Title/Abstract])) OR (“Double blind method” [Title/Abstract])) OR (“crossover procedure” [Title/Abstract])) OR (“Cross-over studies” [Title/Abstract])) OR (intervention [Title/Abstract])	1,351,025
3.	((((((((((“folic acid” [MeSH Terms]) OR (“pteroylpolyglutamic acids” [MeSH Terms])) OR (Tetrahydrofolates [MeSH Terms])) OR (Formyltetrahydrofolates [MeSH Terms])) OR (“folic acid” [Title/Abstract])) OR (“pteroylpolyglutamic acids” [Title/Abstract])) OR (Tetrahydrofolate [Title/Abstract])) OR (“vitamin B9” [Title/Abstract])) OR (Formyltetrahydrofolate [Title/Abstract])) OR (Methylfolate [Title/Abstract])) OR (“L-methylfolate” [Title/Abstract])	50,208
4.	#1 AND #2 AND #3	249

**Table 3:** Search strategy for Embase.

	Embase	Results
1.	dementia:ti,ab,kw OR ‘multiinfarct dementia’:ti,ab,kw OR ‘frontotemporal dementia’:ti,ab,kw OR ‘alzheimer disease’:ti,ab,kw OR ‘disorders of higher cerebral function’:ti,ab,kw OR amnesia:ti,ab,kw OR ‘degenerative disease’:ti,ab,kw OR ‘cognitive defect’:ti,ab,kw OR ‘cognitive impairment no dementia’:ti,ab,kw OR ‘lewy body’:ti,ab,kw OR cognition:ti,ab,kw	330,924
2.	‘clinical trial’:ti,ab,kw OR ‘controlled study’:ti,ab,kw OR ‘randomized controlled trial’:ti,ab,kw OR ‘controlled clinical trial’:ti,ab,kw OR ‘double blind procedure’:ti,ab,kw OR ‘crossover procedure’:ti,ab,kw OR trial:ti,ab,kw OR intervention:ti,ab,kw	1,856,599
3.	‘folic acid’:ti,ab,kw OR pteroptin:ti,ab,kw OR ‘tetrahydrofolic acid derivative’:ti,ab,kw OR ‘vitamin b9’:ti,ab,kw OR methylfolate:ti,ab,kw	30,177
4.	#1 AND #2 AND #3	192

**Table 4:** Search strategy for lilacs.

	LILACS	Results
1.	(mh:(dementia)) OR (mh:(Alzheimer Dementia)) OR (mh:(Alzheimer Dementias)) OR (mh:(Alzheimer Type Dementia (ATD))) OR (mh:(Alzheimer Type Dementia Senile Dementia)) OR (mh:(Dementia, Frontotemporal)) OR (mh:(Dementia, Frontotemporal Lobe)) OR (mh:(Dementia, Lacunar)) OR (mh:(Dementia, Mixed)) OR (mh:(Dementia, Primary Senile Degenerative)) OR (mh:(Dementia, Senile)) OR (mh:(Dementia, Subcortical Vascular)) OR (mh:(Dementia, Vascular)) OR (mh:(Dementias, Frontotemporal)) OR (mh:(Dementias, Frontotemporal Lobe)) OR (mh:(Dementias, Lacunar)) OR (mh:(Dementias, Subcortical Vascular)) OR (mh:(Dementias, Vascular)) OR (Cognitive Impairments) OR (Dementia) OR (Alzheimer Dementia) OR (Alzheimer Dementias) OR (Dementia, Frontotemporal) OR (Dementias, Lacunar) OR (Impairments, Cognitive) OR (Cognitive Impairments, Mild) OR (Impairments, Mild Cognitive) OR (Mild Cognitive Impairments) OR (Cognitive Disorders) OR (Dementia, Cognitive Disordes)	15.558
2.	(mh:(Clinical Trial)) OR (mh:(Double Blind Method)) OR (mh:(Cross-over Studies)) OR (Randomized Controlled Clinical Trial) OR (Randomized Clinical)	9.686
3.	(mh:(Folic Acid)) OR (mh:(Pteroylpolyglutamic acids)) OR (mh:(Vitamin B9)) OR (Folic Acid) OR (Pteroylpolyglutamic acids) OR (Vitamin B9)	947
4.	#1 AND #2 AND #3	151

**Table 5:** Search Strategy for Scopus.

	Scopus	Results
1.	(TITLE-ABS-KEY (dementia) OR TITLE-ABS-KEY (“dementia vascular”) OR TITLE-ABS-KEY (“frontotemporal dementia”) OR TITLE-ABS-KEY (“alzheimer disease”) OR TITLE-ABS-KEY (“neurocognitive disorders”) OR TITLE-ABS-KEY (“cognition disorders”) OR TITLE-ABS-KEY (amnesia) OR TITLE-ABS-KEY (“neurodegenerative diseases”) OR TITLE-ABS-KEY (“neurodegenerative disease”) OR TITLE-ABS-KEY (“cognitive dysfunction”) OR TITLE-ABS-KEY (“cognitive impairments”) OR TITLE-ABS-KEY (“alzheimer dementia”) OR TITLE-ABS-KEY (“alzheimer’s disease”) OR TITLE-ABS-KEY (“vascular dementia”) OR TITLE-ABS-KEY (“Lewy body disease”) OR TITLE-ABS-KEY (“Lewy body dementia”) OR TITLE-ABS-KEY (“Cognitive function”) OR TITLE-ABS-KEY (cognition) OR TITLE-ABS-KEY (“cognitive symptoms”) OR TITLE-ABS-KEY (“cognitive symptom”))	890,582
2.	(TITLE-ABS-KEY (“clinical trial”) OR TITLE-ABS-KEY (“randomized controlled trial”) OR TITLE-ABS-KEY (“Controlled Clinical Trial”) OR TITLE-ABS-KEY (“controlled trial”) OR TITLE-ABS-KEY (trial) OR TITLE-ABS-KEY (“double blind procedure”) OR TITLE-ABS-KEY (“double blind method”) OR TITLE-ABS-KEY (“crossover procedure”) OR TITLE-ABS-KEY (“Cross-over studies”) OR TITLE-ABS-KEY (intervention))	4,040,072
3.	(TITLE-ABS-KEY (“folic acid”) OR TITLE-ABS-KEY (“pteroylpolyglutamic acids”) OR TITLE-ABS-KEY (tetrahydrofolate) OR TITLE-ABS-KEY (“vitamin B9”) OR TITLE-ABS-KEY (formyltetrahydrofolate) OR TITLE-ABS-KEY (methylfolate) OR TITLE-ABS-KEY (“L-methylfolate”))	86,427
4.	#1 AND #2 AND #3	1221

## Data Availability

Not applicable.
